# E2025, a novel anti‐EphA4 antibody, enhances EphA4 cleavage, and suppresses tau pathologies in a transgenic model of AD

**DOI:** 10.1002/alz.094810

**Published:** 2025-01-09

**Authors:** Eiji Inoue, Akio Yamada, Maki Deguchi, Ikuko Yamamoto, Yoshihisa Arita, Aki Nakatani, Tomomi Kawakatsu, Ryunosuke Nakatani, Yoshimasa Sakamoto

**Affiliations:** ^1^ Eisai Co., Ltd., Tsukuba, Ibaraki Japan; ^2^ Eisai Co., Ltd., Kobe, Hyogo Japan

## Abstract

**Background:**

Synaptic degeneration is characteristic of neurodegenerative diseases. Amyloid‐beta (Aβ) plaques and neurofibrillary tangles of hyperphosphorylated tau are known to induce the synapse pathologies directly or indirectly in Alzheimer’s disease (AD). EphA4 is a member of the ephrin receptor subfamily which is predominantly expressed in the brain. Activation of the EphA4 is involved in neurodegeneration in several neurological diseases, including AD. E2025 is a humanized anti‐EphA4 antibody specifically binding to EphA4. We provide evidence that our novel anti‐EphA4 antibody E2025 suppresses neurodegeneration by modulating the EphA4 via direct binding to EphA4.

**Method:**

E2025 was characterized in several in vitro assays for evaluating binding property of E2025 to EphA4, receptor subfamily specificity and potential to inhibit ligand binding. The pharmacological effects of E2025 were assessed using rat primary cultured hippocampal neurons. The in vivo effects of E2025 were investigated in human Tau P301L transgenic mice (Tau tg mice) by immunohistochemical analysis.

**Result:**

E2025 specifically bound to EphA4 with high affinity, inhibiting binding of its ligand to EphA4. In rat primary cultured neurons, E2025 enhanced cleavage of EphA4. In vivo experiments demonstrated that repeated injection of mouse surrogate antibody of E2025 significantly suppressed tau pathology measured by anti‐phosphorylated tau (AT8) antibody.

**Conclusion:**

E2025 specifically suppresses the EphA4 pathway via multiple modes of action, and suppresses progression of tau pathology in a transgenic tau model of AD.

